# Serum Ferritin: A Backstage Weapon in Diagnosis of Dengue Fever

**DOI:** 10.1155/2017/7463489

**Published:** 2017-10-02

**Authors:** Soumyabrata Roy Chaudhuri, Subhayan Bhattacharya, Mainak Chakraborty, Kingshuk Bhattacharjee

**Affiliations:** ^1^Woodlands Hospital, 8/5 Alipore Road, Alipore, Kolkata West Bengal 700027, India; ^2^Department of Tropical Medicine, School of Tropical Medicine, 108 Chittaranjan Avenue, Kolkata, West Bengal 700073, India; ^3^Medical Services, Biocon Limited, Bengaluru 560100, India

## Abstract

**Aims:**

This retrospective study evaluates ferritin as a surrogate marker for dengue infection (NS1 and IgM negative stage) as opposed to other febrile illnesses of infective or inflammatory etiology (OFI).

**Methodology:**

Data of all patients admitted to medical ward and medical ITU during the dengue outbreak were collected. Patients admitted between 5 and 10 days of febrile illness without a diagnosis were included. Patients with NS1 positivity (Days 2–8) and/or positive IgM for dengue (Days 6–10) were considered to be dengue cases and those with other confirmed diagnoses were considered in the OFI group. Ferritin, CRP, TC of WBC, platelet count, SGOT, SGPT, and albumin levels were analysed for both groups.

**Results:**

We examined 30 cases of clinically and serologically confirmed dengue fever and 22 cases of OFI. Ferritin level in dengue cohort was significantly higher than the OFI group (*p* < 0.0001). The best cut-off for ferritin level to differentiate dengue from OFI was found to be 1291. The sensitivity at this cut-off is 82.6% and the specificity at this cut-off is 100%.

**Conclusion:**

Ferritin may serve as a significant marker for differentiating between dengue fever and OFI, in absence of a positive NS1 antigen or a positive IgM antibody for dengue.

## 1. Introduction

Dengue fever is one of the world's important viral hemorrhagic fevers, most geographically widespread of the arthropod-borne viral illnesses, caused by* Arbovirus* of* Flavivirus* genus with 4 serotypes [1, 2], and affects an estimated 3.97 billion people across 128 endemic countries including India [3]. It is transmitted by* Aedes aegypti* and* Aedes albopictus* mosquitoes. Four spectra of illness are seen: an asymptomatic phase, acute febrile illness, classic dengue fever with or without hemorrhagic manifestation, and dengue hemorrhagic fever (DHF) which includes Dengue Shock Syndrome (DSS) and expanded dengue syndrome [4]. Clinically dengue fever is suspected when acute febrile illness of 2–7 days presents with two or more than two of the following, namely, headache, retroorbital pain, myalgia, arthralgia, rash, and hemorrhagic manifestations [5]. Dengue fever is diagnosed by NS1 antigen reactivity by ELISA method usually for the first 5 days of fever. After that IgM detection by MAC-ELISA is used to diagnose dengue fever but IgM appears usually within 5–7 days of fever but sometimes it may take more time, even up to 12 days, to appear [6]. Detection of NS1 antigen is a fair tool for diagnosing dengue virus infection (DVI). The sensitivity of NS1 for diagnosis is more than 90% within 2-3 days of illness. But the sensitivity gradually decreases after that period and it is even lower beyond 5th day [7]. Detection of dengue virus specific IgM can also diagnose DVI with a good sensitivity and specificity. In patients not previously infected with dengue virus, this IgM response is slow rising. It is 50% in 3–5 days, 80% in more than 5 days, and 99% in 10th day [8]. Furthermore, IgM dengue antibody may be nondetectable till 8th day of illness. In secondary DVI IgM response is much blunted and it appears much later in the timeline. IgG in that case appears earlier than IgM. NS1 disappears from blood much early in secondary DVI due to presence of neutralizing antibody [9]. Under these circumstances we find many cases where after cessation of NS1 response IgM was yet to appear. In those cases we find raised serum ferritin is a surrogate for diagnosis but never confirmatory.

In dengue fever, serum ferritin is disproportionately raised compared to any bacterial or viral infection and this elevated level corroborates with an increased risk of developing complications. Some studies showed a very strong correlation between serum ferritin level and severity of dengue infection [10]. Again serum ferritin measured on 4th or 5th day roughly evaluates the prediction of dengue infection [11].

A study from the Caribbean island Aruba concluded that ferritin can be used as a clinical marker to discriminate between dengue and other febrile illnesses [12]. The occurrence of hyperferritinemia in dengue virus infected patients is indicative for highly active disease resulting in immune activation and coagulation disturbances. Therefore, patients with hyperferritinemia are recommended to be monitored carefully. The same study concluded that high serum ferritin level with a cut-off value of >1500 in confirmed DENV infection is associated with increased severity of dengue related illness in adults. Ferritin levels measured at Day 4 or 5 may be a good predictor in outcome in dengue [11].

## 2. Aims and Objective 

This retrospective study was aimed at evaluating whether elevated levels of ferritin could serve as a surrogate marker of DENV infection, rather than in other febrile illnesses (OFI) of other infective or inflammatory etiology.

### 2.1. Materials and Method

Our retrospective study looked at the database of a Kolkata based (a metropolis of eastern India) corporate multispecialty hospital. Data of all patients admitted to medical ward and medical ITU during the months of September and October 2016 (a period when dengue incidence was on the high) were collected and analysed. Patients admitted with undiagnosed cause of fever, in whom ferritin, CRP, TC of WBC, platelet count, SGOT, SGPT, and albumin levels (any one of the seven parameters) were not measured, were excluded from the analysis. Patients with NS1 positivity (Days 2–8) and/or positive IgM for dengue (Days 6–10) prior to discharge were considered to be dengue cases. We have excluded data of all the patients in whom either NS1 or IgM was positive within Day 7 from the time of admission.

Dengue IgG and IgM were estimated by micro ELISA kit (J Mitra) with Roche 9180 autoanalyser and NS1 positivity was estimated by ELISA kit (J Mitra) with Reader Washer P40PR4100. Ferritin was estimated by ECLIA (Hitachi) with Roche Cobas E411. LFT was estimated by Dry Chemistry System (Ortho Clinical Diagnostics) with Vitros 5.1/FS. Hb, TC, DC, and platelet were estimated by autoanalyser (Transasia) with autoanalyser KX-21 XT1800i.

In febrile patients due to other etiology, absence of a biochemical, hematological, microbiological, or radiological definitive diagnosis leads to their exclusion. None of the patients below 18 years or above 65 years were taken up for the analysis.

### 2.2. Statistical Methods

Descriptive statistical analysis has been carried out in the present study. Results on continuous measurements are presented on mean ± SD and results on categorical measurements are presented in numbers (%). Significance is assessed at a level of 5%.

The following assumptions on data are made. Normality of data was tested by Anderson Darling test, Shapiro-Wilk test, and Kolmogorov-Smirnoff test and visually by QQ plot. Mann–Whitney *U* test or unpaired *t*-test has been used to find the significance of study parameters between two groups of patients. ROC analyses were performed to find the significant predictor of dengue. We could not use discriminant analysis to evaluate ferritin levels as a discriminating factor because ferritin was not normally distributed, thereby violating the basic assumption of discriminant analysis.

### 2.3. Statistical Software

The statistical software, namely, SAS (Statistical Analysis System), version 9.2 for Windows, SAS Institute Inc., Cary, NC, USA, and Statistical Package for Social Sciences (SPSS Complex Samples) Version 21.0 for Windows, SPSS, Inc., Chicago, IL, USA, were used for the analysis of the data. Microsoft Word 2010 and Microsoft Excel 2010 (Microsoft Corp., Redmond, WA, USA) have been used to generate tables (Lines #101–107).

## 3. Results

Out of 358 cases of serological proven dengue, this study examined only 30 cases (*N* = 30, males = 17 and females = 13 with a mean age of 39.86 ± 12.95) of confirmed dengue fever which was proved clinically as well as serologically by dengue IgM reactivity beyond 7th day of febrile illness (all these 30 patients tested negative for NS1 antigen). In our retrospective cohort of 30 cases, 28 patients were admitted during 4th to 7th day of their illness and remained NS1 nonreactive as well as negative for IgG-M till 7th day. The remaining two patients were NS1 negative cases who were admitted in late hours of 7th day of their febrile illness. All patients became positive for IgM dengue antibody on 8th day of illness except one out of the two 7th-day late hour admission. This patient tested positive for IgM antibody that appeared on 9th day.

According to the protocol of dengue management, patients were managed conservatively and relevant investigations were done. We also noted reports of another 22 patients (*N* = 22, males = 14 and females = 8 with a mean age of 48.09 ± 16.72) of known diseases presenting with short term fever within 7 days of onset in whom diagnosis of dengue was excluded by establishing other confirmed diagnosis.

The values of different parameters including the acute phase reactants are as enlisted in Table 1.

Ferritin level in the dengue cohort (median 2745, IQR 1574–3452) was significantly higher than the nondengue cohort/other febrile illness (OFI) (median 344.15, IQR 157–815.2), *p* < 0.0001 as computed by Mann–Whitney test. Median CRP level in the dengue cohort (18.2) was significantly lower than the OFI cohort (47.55), *p* = 0.040. TLC (total leukocyte count) value in the dengue cohort (3300) was significantly lower than that of OFI cohort (8250), *p* = 0.0001, and so was the platelet count (median 60000, IQR 35000–140000, versus median 182500, IQR 140000–230000). No significant difference in the albumin levels was noted. Both SGPT (median value 101) and SGOT (median value 118) were significantly higher than the OFI cohort (344.15), *p* = 0.0003 and *p* = 0.002, respectively.

**Figure 1:**
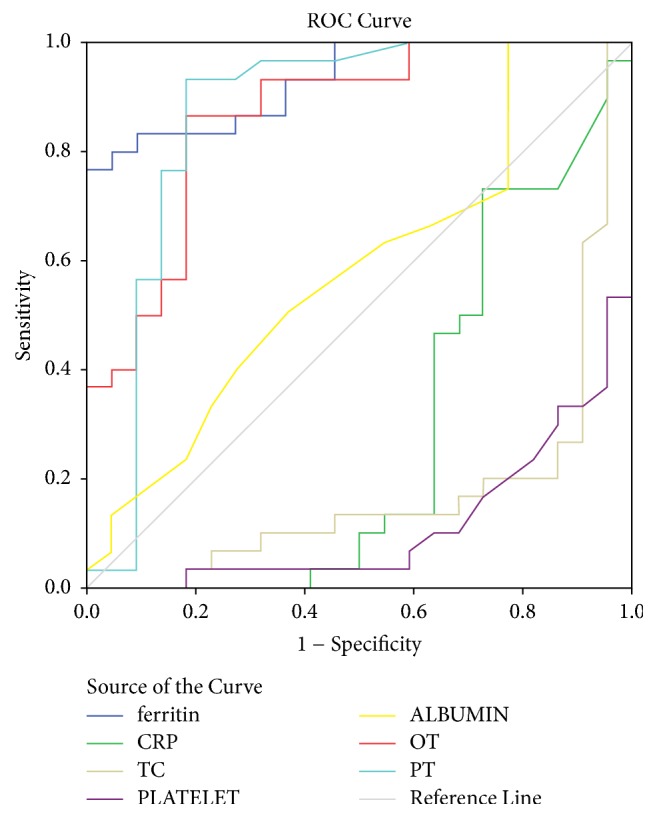
Receiver operating characteristic curve for identifying discriminating predictors of dengue. Diagonal segments are produced by ties.

The area under the curve as obtained by ROC analysis for ferritin in predicting the dengue versus nondengue is 0.942 with 95% confidence interval (.874, 1.00) (Table 2, Figure 1). The accuracy of the test depends on how well the test separates the group being tested into those with and without the disease in question. Accuracy is measured by the area under the ROC curve. An area of 1 represents a perfect test; an area of .5 represents a worthless test. A rough guide for classifying the accuracy of a diagnostic test is the traditional academic point system: 0.90–1 = excellent (A), 0.80–0.90 = good (B), 0.70–0.80 = fair (C), 0.60–0.70 = poor (D), and 0.50–0.60 = fail (F). The AUC is 0.942 with 95% confidence interval (.861, 1.00). Thus we can say that ferritin level was found as a good to excellent predictor in the diagnosis of dengue with regard to differentiating from the nondengue group.

The best cut-off for ferritin level to differentiate dengue from OFI was found to be 1291. The sensitivity at this cut-off is 82.6% and the specificity at this cut-off is 100%.

## 4. Discussion

Dengue fever is a dynamic febrile illness that can range from a mild self-limiting form to the other end of the spectrum which ranges from plasma leakage, haemorrhage, or severe multiorgan dysfunction leading to severe life threatening situation. An array of mechanisms have been proposed to explain the pathogenesis of severe dengue that includes antibody-dependent enhancement [ADE] of viral infection [13, 14], overwhelming activation of memory T cell [15], and proinflammatory cytokine response exaggeration [16, 17] which leads to the often fatal dengue hemorrhagic fever [DHF] and Dengue Shock Syndrome [DSS] [18–20].

In recent times a phenomenon called macrophage activation syndrome (MAS) or hemophagocytic syndrome (HS) is being frequently reported in patients with severe dengue. MAS is a severe systemic inflammatory condition due to excessive activation and proliferation of T cells and well differentiated macrophage that leads to hyperactivated but dysregulated immune responses. This results in an overwhelming inflammatory response leading to nonremitting high rise of temperature, organomegaly (involving liver and spleen), hemorrhage, lymphadenopathy, and central nervous system (CNS) dysfunction [21]. Hyperferritinemia (levels above 10000 ug/L) is a flagship sign of MAS; however hypoalbuminemia, cytopenia, coagulopathy, abnormal liver function tests, hypertriglyceridemia, hemophagocytosis, and elevated serum sCD25 and sCD16 levels also serve as adjunct markers of MAS [21–25]. Hemophagocytic syndrome (HS) is being increasingly reported in patients with severe dengue with multiorgan complication [26] and is observed in severe dengue involving children as well as adults [27–29] and is notably associated with dyserythropoiesis [30].

During the course of febrile illness, there is a window period during which both NS1 antigen and IgM antibody for dengue may be negative and also biochemical or hematological parameters pointing towards other alternative diagnosis are absent too. In this period of dilemma as to the cause of the febrile illness, clinicians are at a loss with regard to the choice of an acceptable line of management for the said state. Successful management of dengue fever is by early diagnosis and optimal fluid resuscitation with an aim of correcting or of preventing onset of dehydration and thereby reducing chances of complications as discussed above. Hence there arises a need to look for other surrogate markers which will tell the tell-tale story of febrile illness due to DENV infection even if NS1 is negative and IgM antibodies for dengue are yet to appear.

This study retrospectively analysed 30 proven cases of dengue fever versus 22 cases of short term febrile illness, in whom there was an established diagnosis as to the cause of fever apart from dengue (OFI). The OFI subgroup had diagnoses of varied etiology ranging from* Salmonella typhi*, malaria, and Koch's infections to inflammatory fevers from acute flair of rheumatoid arthritis (RA).

The present study looked at the differential behavior of serum ferritin in the patients of DVI and the patients not infected with dengue. Hence the control group was comprised of patients with acute febrile illness in whom DVI was satisfactorily excluded. In our study, we therefore grouped those patients to the control arm who had no laboratory signs of DVI for a substantial period of time. However, we had to exclude our observations for a number of times because the patient left the hospital against medical advice or was admitted beyond 10th day of the febrile illness (due to the time window of IgM dengue positivity).

Mean ferritin level in the dengue subgroup was 3492.56 and median was 2745 with IQR (interquartile range) 1574–3452 whereas, in the OFI group, mean ferritin level was 470.01 and median was 344.15 with IQR 157–815.2; *p* value < 0.0001 was statistically significant. ROC analysis for assessing ferritin as a diagnostic marker in the dengue versus OFI subset revealed area under the curve to be 0.942 with a standard error of .035, *p* value of <0.0001 (95% confidence interval .874–1.00) which implies that ferritin is a good to excellent differentiator between the dengue and OFI group. The best cut-off level for ferritin was 1291 with a sensitivity of 82.6% and a specificity of 100%. This however is a little different from the findings of Ho et al. [12] who reported a sensitivity of 44% and a specificity of 88%. This difference can be well explained by the fact that the current study included retrospective analysis of hospitalized patients only containing more of severe dengue cases (patients with shock, respiratory distress, severe bleeding, and/or organ impairment) or patients of nonsevere dengue with warning signs (WS+- characterized by abdominal pain, vomiting, minor mucosal bleeding, pleural effusion, ascites, and hepatomegaly) whereas the study conducted jointly by the Brazilian and Dutch Medical scientists looked at a rather mild epidemic between September 2011 and April 2012 wherein only one case of severe dengue was recorded according to the WHO 2009 dengue case classification.

The current retrospective study also looked at C-reactive protein (CRP) and its differences between the dengue and the OFI group. The mean CRP in the dengue subgroup was 19.77, with a median of 18.2 and IQR ranging from 5 to 27.1 whereas the OFI group had a much higher CRP with a mean of 105.50, median of 47.55, and an IQR ranging from 6.3 to 189 and a *p* value of 0.040, which achieved statistical significance. However the ROC analysis proved that CRP was not a suitable distinguishing marker between the two groups as the area under the curve (AUC) was 0.310 much lesser than 0.5 mark (reference line) which would classify it as a distinguishing marker. Total count of WBC and platelet count were also lower in the dengue group rather than in the OFI group and both attained statistical significance (*p* = 0.0001 and *p* = 0.0002, resp.) but when subjected to ROC analysis, both failed to produce a significant area under the curve (AUC) and thus failed to achieve significance as a distinguishing marker between the two subsets of patients.

Three other parameters were also studied in this small retrospective study, namely, albumin, SGOT, and SGPT. However albumin which is a well-known acute phase reactant had a mean of 3.46, median of 3.5, and IQR range of 3.1–3.8 in the dengue subset whereas in the OFI subgroup it had a mean of 3.28, median of 3.4, and an IQR range of 3.2–3.7 and thus failed to achieve statistical significance in the Mann–Whitney test (*p* = 0.51). Albumin thereby did not show any significance as a marker to differentiate between the two groups of febrile illness. SGOT and SGPT achieved statistical significance in the Mann–Whitney test and were thus taken up for ROC analysis where the AUC for SGOT was 0.870 and for SGPT was 0.803 and thus both qualified as significant distinguishing markers between the dengue and OFI group. However hyperferritinemia in dengue fever is associated with elevation of both SGOT and SGPT, as reported in the paper on DENV infection in the Aruba Islands by the Brazilian and the Dutch medical researchers [12]. A study conducted by Itha et al. showed that 43 out of 45 patients with DENV infection had elevated levels of SGOT and SGPT [30] and Chhina et al. [31] also showed that elevation of liver enzymes secondary to hepatic dysfunction was common in all forms of dengue infection with a preferentially high SGOT than SGPT being found in 90% of the patients with dengue fever. Moreover the absence of hepatic infections in the OFI subset also contributed to the lower SGOT and SGPT in this group. Therefore, although the hepatic enzymes did present themselves as a good differentiating marker between the two subsets of febrile patients, it is to be remembered that such values of SGOT and SGPT may also be encountered in nondengue fever (OFI) where the primary focus of infection is in the liver (namely, viral hepatitis, liver abscess, etc.).

## 5. Limitations

The study is a retrospective analysis involving small number of dengue ad nondengue febrile illnesses (30 and 22, resp.). The OFI group did not have subjects with primary focus of infection in the liver. Elevated SGOT and SGPT in such patients would have posed a challenge in the evaluation of the enzymes in the two subgroups. The spectrum of dengue infection is wide and this study only looked at the hospitalized patients with severe dengue and also at admitted patients with nonsevere dengue having warning signs positive. However, a large set of patients with nonsevere dengue treated on an OPD basis are not captured in this study. A major limitation is that we have conducted the study with a relatively small number of samples. However this data adds to the existing data of medical literature and recommends extending our study during future dengue outbreaks.

## 6. Conclusion

Dengue fever has various manifestations at presentation ranging from mild febrile state to life threatening forms, namely, DSS, MAS, and/or HS. During the course of febrile illness, there often arises a time frame where neither the NS1 antigen is positive, nor have the IgM antibodies for dengue appeared. In this period of dilemma in the clinicians' front, ferritin was evaluated as an adjunct marker for the diagnosis of dengue which could possibly aid their clinical judgment and prompt early fluid resuscitation which in turn could be useful in avoiding undue complications. Ferritin, as evaluated in the present study may serve as a significant marker for differentiating between dengue fever and fever of other etiology, even in the absence of a positive NS1 antigen or a positive IgM antibody for dengue.

However, prospective trials involving larger numbers are needed to further strengthen the evidence base in favour of ferritin before it can undoubtedly be used as another marker to distinguish between dengue fever and fever due to other illness.

## Figures and Tables

**Table 1:** Comparative analysis of pertinent parameters for the dengue and nondengue cohort.

	Dengue, *N* = 23				Nondengue, *N* = 18				
Label	Mean	SD	Median	Interquartile range	Mean	SD	Median	Interquartile range	*p*
Ferritin, ng/mL	3492.56	3282.96	2745	1574–3452	470.01	351.96	344.15	157–815.2	<0.0001
CRP, mg/L	19.77	16.17	18.2	5–27.1	105.50	131.24	47.55	6.3–189	0.040
TC, number per cumm	4244.78	2879.40	3300	2500–4400	9497.22	4526.15	8250	6200–14100	0.0001
Platelet, number per cumm	87130.43	70978.42	60000	35000–140000	200444.44	101601.43	182500	140000–230000	0.0002
Albumin, gm/dL	3.46	0.39	3.5	3.1–3.8	3.28	0.61	3.4	3.2–3.7	0.51
SGOT, U/L	200.5	175.5	121.5	98–219.75	63.50	45.62	52	29.29–84.25	0.001
SGPT, U/L	130.53	99.6	107	65–159	62.55	91.00	32	25–45	0.015

*p* < 0.05 considered as statistically significant and *p* computed by Mann–Whitney test or unpaired *t*-test.

**Table 2:** Area under the curve for different parameters by receiver operating characteristic curve.

Test result variable(s)	Area	Std. error	Asymptotic 95% confidence interval of the area under the curve	
			Lower bound	Upper bound
Ferritin	.942	.035	.874	1.000
CRP	.310	.091	.132	.489
TC	.114	.054	.008	.219
Platelet	.127	.055	.020	.234
SGOT	.870	.058	.756	.984
SGPT	.803	.079	.648	.958

## Additional Points

*Key Messages*. Ferritin, as evaluated in the present study, may serve as a significant marker for differentiating between dengue fever and fever of other etiology, even in the absence of a positive NS1 antigen or a positive IgM antibody for dengue.
